# On the Reparative Power of the Spinal Cord after Complete Division

**Published:** 1852-07

**Authors:** E. Brown-Sequard

**Affiliations:** Paris


From the Medical Examiner.

*On the Reparative Power of the Spinal Cord after complete Division.* By E. Brown-Sequard, M.D., of Paris.

I have performed a large number of experiments, with the view of determining the degree of curability of wounds of the spinal marrow. When I began these researches, I believed, with every physician, that exposure of the spinal cord to the action of the air, was an extremely dangerous operation. I had read and accepted as true, the following assertion of Dr. Longet: "All the experimenters who have had an opportunity of opening the vertebral canal on adult animals of the higher classes, ought to know that as soon as the medulla spinalis, *even when surrounded by its liquid and by the dura mater*, has been laid bare, in the lumbar region, the nervous action becomes so much enfeebled, that the animals are unable to keep themselves on their legs, which at the same time appear to be quite insensible. Suddenly, after the dura-mater has been cut, and the cerebro-spinal liquid has escaped through the wound, the state of the animal becomes worse; it falls down, struck by paralysis, and the posterior extremities may be cut without exciting the least appearance of pain."*

I have found that this description of phenomena is true only in certain cases, and in certain animals, (as the dog.) When the operation is performed slowly, and so as to give much pain to the animal, and produce a considerable hæmorrhage, then it happens that an appearance of palsy follows the laying bare of the cord; but, even in that case, if the animal is left quiet for a short time, it recovers, and sensibility, together with voluntary motion, return in his hind limbs.

When the operation is performed upon cats, sheep and guinea-pigs, there is usually no appearance of palsy; but when it is greatly prolonged, it happens sometimes that, in consequence of the extreme exhaustion produced by the hæmorrhage, and by excess of pain, the animal becomes apparently paralyzed, even before the spinal canal has been opened, so that it is not the action of air on the spinal marrow which causes the apparent palsy.

My experiments have gone so far as to prove that mammals may not only have no appearance of palsy, after opening their spinal canal, but that they are able to live long and apparently in very good health after their medulla spinalis has been exposed to the action of the air. I have seen guinea-pigs living very well after I had taken out eight or ten posterior arches of the lumbar and sacral vertebræ. There has been only a slight disturbance in the movements of the posterior limbs of these animals, and that disturbance, very probably, was in consequence of the excision of the muscles inserted upon the vertebral column.

After having stated the innocuity of the action of air on the spinal marrow, I have tried experiments on wounds of that organ.

It is known that there are clinical observations proving that the injuries of the spinal cord are not constantly followed by death, and that the paraplegia which is the result of these wounds may disappear more or less completely; but there is no observation proving the possibility of an entire curation i.e., of a full return of sensibility and of voluntary motion after the complete transverse section of the spinal cord. The celebrated experiments of Arne-

* Traite d'Anat. et de Physiol. du Syst. Nerv., 1842. T. 1, p. 276.

mann, Flourens, Ollivier (d'Angers) and Jobert (de Lamballe) have left the question of the possibility of recovery after complete transverse section of the medulla spinalis heretofore unsettled.

My first experiments, made upon pigeons, had shown me an incipient return of the lost functions some months after the complete section of the spinal cord. I afterwards saw five pigeons, upon which such a section had been made, gaining little by little, and at last entirely, both sensibility and voluntary motion. I have not been able to examine the spinal marrow of all these pigeons; but upon three of them the most careful microscopical examination of the injured cord has been made. One of these three animals had been operated upon eighteen months before the anatomical examination. The history of this pigeon is very interesting. Its spinal cord was *entirely divided* transversely between the fifth and sixth costal vertebræ. The operation was followed by complete paralysis of the posterior part of the body, both as regarded sensibility and voluntary motion. At the end of three months, voluntary movements began to show themselves, in connection with reflex actions; and sensibility appeared to exist anew. These powers gradually augmented; and six months after the operation, the bird could stand for some minutes, but fell as soon as it attempted to walk. In the course of the seventh month it began to walk, but unsteadily, helping itself constantly with its wings. By the end of the eighth month, it could walk slowly without support; but if it attempted to walk fast, it fell over, unless it supported itself with its wings. When it was walking a little faster than usual, it loosened its wings a little, so as to be ready to prevent itself from falling. The beginning of the thirteenth month, it could run. Fifteen months after the operation, its progression seemed in all respects normal, save that a certain degree of stiffness remained in its gait. The generative function also, which had been entirely destroyed by the operation, was completely restored. It was a male.

My friend, Dr. Ch. Robin, assisted me in the autopsy of that pigeon. We found a bundle of cellular fibres uniting the dura mater to a part of the spinal cord where a whitish, opaque circular line existed. At that place the cord was somewhat contracted, its transverse diameter being smaller than elsewhere. A very fine longitudinal slice of the cord taken from the place of contraction, and examined with the microscope, showed us a great number of normal double and single-edged nervous tubes. There was nothing peculiar in that cicatrix, except; 1st, that the nerve-fibres exhibited to a greater degree than usual, those varicosities which are found in nerve-fibres of the soft parts of the brain and of the cerebral nerves; 2d, that the nervous corpuscles, instead of being only in the central part of the cord, were scattered every where amidst the nerve-tubes.

In two other cases, the microscopical examination having been made by my friends Dr. Lebert and Dr. Folling, we found a like reunion of the nervous fibres of the cord.

In several guinea-pigs, in which I had made the section through only one-half of the spinal cord, an incomplete return of voluntary power was observed within seven or eight months after the operation. In the case of one of these, a guinea-pig which had been subjected to this operation the year before, and in which sensibility appeared to have been completely restored, and voluntary movement less completely, I made, with my friend M. Laboulbere, a careful examination of the injured part. We found that the section had traversed both the posterior columns, as well as the anterior and lateral columns, and a portion of the gray substance on the right side, all of which parts exhibited a sort of contraction, the continuity of the divided parts being re-established by a whitish opaque cicatrix. On examining the substance of this cicatrix we found it in part made up of fibres of areolar tissue, the direction of which was transverse or very slightly oblique. These cellular fibres were crossed by great numbers of normal double-edged nerve-fibres running in a longitudinal direction. The nerve-fibres were found uninterruptedly continuous not only through the whole extent of the cicatrix, but also before and behind. There were a small number of nerve-corpuscles scattered between the nerve and areolar fibres.

From these researches I draw the following conclusions:

1st, That the spinal marrow, even in adult mammalia, may be exposed to the action of the air without danger to the life of the animal.

2d, That wounds of the spinal marrow may be repaired.

3d, That after a complete transverse section of the spinal cord, the functions of that organ may be entirely restored.

